# Trends in HIV detection, AIDS detection, and ADIS mortality in Tocantins, Brazil, 2015–2023: Impact of the COVID-19 pandemic

**DOI:** 10.1590/1980-549720260050

**Published:** 2026-08-28

**Authors:** Orcélia Pereira Sales, Edgar Merchan-Hamann

**Affiliations:** I Universidade de Brasília, Graduate Program in Health Sciences – Brasília (DF), Brazil.

**Keywords:** HIV, AIDS, Time Series Studies, COVID-19, Epidemiology

## Abstract

**Objective:**

To analyze the temporal evolution of epidemiological indicators related to HIV/AIDS in the state of Tocantins between 2015 and 2023 and to assess potential changes associated with the COVID-19 pandemic.

**Methods:**

An ecological time-series study was conducted using secondary data from national health information systems. Annual rates per 100,000 inhabitants were calculated. Trends were estimated using Prais–Winsten regression, and pandemic-associated changes were assessed using interrupted time-series analysis.

**Results:**

Between 2015 and 2023, 1,854 HIV cases, 2,026 AIDS cases, and 511 AIDS-related deaths were recorded in Tocantins. The mean annual rates were 13.09 HIV cases, 14.32 AIDS cases, and 3.61 AIDS-related deaths per 100,000 inhabitants. Prais–Winsten analysis indicated stationary trends for HIV detection, AIDS detection, and AIDS mortality rates. In the interrupted time-series analysis, no significant immediate changes were observed after 2020. A significant increase was observed only in the trend of the AIDS detection rate during the post-interruption period (β = 2.67; 95%CI 0.26; 5.09; p=0.036), whereas HIV detection and AIDS mortality rates remained stable.

**Conclusion:**

Trends in HIV detection, AIDS detection, and AIDS mortality rates remained stable in Tocantins between 2015 and 2023. However, an increase in the trend of the AIDS detection rate was observed after the onset of the COVID-19 pandemic, suggesting potential repercussions of this period on the dynamics of disease diagnosis and reporting.

## INTRODUCTION

Human Immunodeficiency Virus (HIV) infection and Acquired Immunodeficiency Syndrome (AIDS) remain among the major public health challenges worldwide, despite decades of advances in diagnosis, treatment, and prevention. Expanded access to antiretroviral therapy and combination prevention strategies have significantly reduced mortality and altered the clinical course of the infection. However, social and structural inequalities continue to influence access to health services and transmission dynamics, resulting in an unequal distribution of the disease burden across populations and territories^1,2^.

In Brazil, the policy of universal and free access to antiretroviral therapy is established as an international benchmark; however, the epidemic continues to exhibit marked territorial and social heterogeneity. Detection and mortality rates vary substantially across regions and federative units, following gradients in income, education, and race/skin color that define different levels of vulnerability. Socioeconomic and structural determinants influence both exposure to risk and timely access to prevention and treatment, contributing to the persistence of inequalities in the response to the epidemic^1–3^.

The Northern region faces additional challenges in addressing HIV/AIDS. Its vast territorial extent, population dispersion, and unequal availability of health services hinder timely diagnosis and continuity of care, with repercussions for trends in detection and mortality. Studies conducted in the Brazilian Amazon have revealed disparities in care trajectories across states, barriers to access to prevention tools such as pre-exposure prophylaxis, and mortality patterns that reflect regional inequalities^4–6^.

The state of Tocantins shares many of the challenges observed in the Northern region, including its vast territorial extent, a population distributed across small municipalities, and inequalities in access to health services^4,5,7^. Nevertheless, few studies have evaluated the temporal trends of two indicators related to HIV/AIDS, particularly those addressing potential changes associated with the COVID-19 pandemic^8,9^. This gap hinders an understanding of the recent dynamics of the epidemic and limits the generation of evidence to inform the planning, monitoring, and evaluation of surveillance, prevention, and care activities.

The COVID-19 pandemic represents an event capable of significantly altering the dynamics of health services and epidemiological surveillance systems. The reorganization of the healthcare network, the prioritization of care for COVID-19 cases, and mobility restrictions and limitations imposed during a public health emergency have repercussions for the provision of testing, timely diagnosis, and clinical support for people living with HIV/AIDS^3,10,11^. Studies conducted in different contexts have documented reductions in diagnoses, delays in linkage to care, and difficulties in accessing specialized services during the first years of the pandemic^10–15^. In Brazil, a substantial reduction in HIV and AIDS notifications was observed in 2020, a phenomenon attributed mainly to reduced testing and changes in the organization of health services^2,4,8^. Considering the territorial and healthcare characteristics of the Northern region^4,5^, it is relevant to investigate the extent to which these changes have affected HIV/AIDS epidemiological indicators in Tocantins.

Although the impacts of the pandemic on the aforementioned indicators have been widely documented at the national and international levels, few studies have analyzed, at the state level, changes in the trends of these conditions, particularly in contexts marked by territorial inequalities, such as Tocantins. The lack of analyses combining trend estimation with the assessment of interruption points limits the understanding of the recent behavior of the epidemic and the identification of potential inflections associated with the pandemic period.

In this context, the present study aimed to analyze the temporal trends of two epidemiological indicators related to HIV/AIDS in the state of Tocantins between 2015 and 2023 and to evaluate potential changes associated with the COVID-19 pandemic.

## METHODS

### Study Design

This was an ecological time-series study^16^, based on secondary data from national health information systems. The study was conducted in accordance with the recommendations of the Reporting of Studies Conducted Using Observational Routinely Collected Health Data (RECORD)^17^ statement, an extension of the STROBE statement for studies that use routinely collected data.

### Background

The state of Tocantins, located in the Northern region of Brazil, has a territorial area of 277,621 km² and a population of 1,511,460 inhabitants, according to the 2022 Demographic Census of the Brazilian Institute of Geography and Statistics (*Instituto Brasileiro de Geografia e Estatística* – IBGE)^18^. The Municipal Human Development Index (MHDI) was 0.731^19^. Administratively, the state is organized into eight health regions and two macroregions, comprising a total of 139 municipalities^20^.

### Data source

Data on HIV cases were obtained from the 2025 HIV/AIDS Epidemiological Bulletin^21^, published by the Ministry of Health, which provides HIV records derived from the integration of databases from the Information System for Notifiable Diseases (*Sistema de Informação de Agravos de Notificação* – Sinan), the Laboratory Test Control System (*Sistema de Controle de Exames Laboratoriais* – Siscel), and the Medication Logistics Control System (*Sistema de Controle Logístico de Medicamentos* – Siclom), according to federative unit of residence and year of diagnosis. Records for the state of Tocantins were extracted for the period from 2015 to 2023. The inclusion of five years before the onset of the COVID-19 pandemic and four subsequent years allowed for greater robustness in the analyses of temporal trends and interrupted time series.

Data on AIDS cases were obtained from Sinan and accessed through the TabNet system of the Department of Informatics of the Brazilian Unified Health System (*Departamento de Informação e Informática do Sistema Único de Saúde* – DataSUS). Data on AIDS-related mortality were obtained from the Mortality Information System (*Sistema de Informação sobre Mortalidade* – SIM), considering deaths for which the underlying cause was classified under codes B20 to B24 of the International Classification of Diseases, 10^th^ Revision (ICD-10). Population estimates used to calculate the rates were obtained from IBGE population projections available through DataSUS.

### Variables

Three main epidemiological indicators were analyzed: HIV detection rates, AIDS detection rates, and AIDS mortality rates.

The HIV detection rate was defined as the number of HIV cases reported in each year of diagnosis per 100,000 inhabitants, according to the methodology adopted by the Ministry of Health in the HIV/AIDS Epidemiological Bulletins.

The AIDS detection rate was defined as the number of newly reported AIDS cases per 100,000 inhabitants, whereas the AIDS mortality rate was defined as the number of deaths for which the underlying cause was classified under ICD-10 codes B20 to B24 per 100,000 inhabitants.

The rates were calculated using the annual number of cases or deaths as the numerator and the estimated resident population for the respective year as the denominator, based on IBGE population estimates.

The absolute numbers of HIV cases, AIDS cases, and AIDS-related deaths reported annually were also described. Additionally, the AIDS case-fatality rate was calculated as the ratio between the number of AIDS-related deaths and the number of AIDS cases reported in the same year, expressed as a percentage.

AIDS case fatality was analyzed exclusively in a descriptive manner and was not included in the temporal trend models, as it represents a ratio between events rather than a population-based rate. Furthermore, this indicator is susceptible to fluctuations resulting from small numbers and to the lack of temporal correspondence between the year of case notification and the year of death.

### Data analysis

A descriptive analysis of the epidemiological indicators was performed, including the calculation of absolute frequencies and annual detection and mortality rates^22^.

Temporal trends in HIV detection, AIDS detection, and AIDS mortality rates were estimated using Prais–Winsten regression, with a base-10 logarithmic transformation of the rates. Trends were assessed based on the estimated coefficients used to calculate the Annual Percent Change (APC), along with the respective 95% confidence intervals (95%CIs) and p-values. Trends were classified as increasing when the 95%CI of the APC was entirely positive, decreasing when it was entirely negative, and stationary when the interval included zero. The presence of serial autocorrelation in the residuals was assessed using the Durbin-Watson statistic.

For the interrupted time-series analysis, the year 2020 was considered the interruption point, representing the onset of the COVID-19 pandemic. The interrupted time-series models included three components: a time variable, used to estimate the pre-interruption trend; a level indicator variable, used to estimate immediate changes after 2020; and an interaction variable between time and the post-interruption period, used to estimate changes in the trend of the series after the onset of the pandemic.

The level variable was coded as 0 for the period before 2020 and 1 for subsequent years. The post-interruption trend variable was constructed by assigning a value of 0 to the years before the pandemic and increasing values to subsequent years, allowing changes in the trend of the series after the interruption to be estimated.

The analyses were performed using R software (version 4.6.0) in the RStudio environment, using the Prais-Winsten package for regression models.

### Considerations regarding bias

As this was an ecological study based on secondary data, the results are subject to underreporting, reporting delays, and differences in the quality of information systems. Changes in diagnostic coverage, service organization, and epidemiological surveillance, particularly during the COVID-19 pandemic, may have influenced the analyzed indicators. Furthermore, the use of aggregate data precludes inferences at the individual level, and the results are therefore subject to ecological fallacy.

### Ethical considerations

The study used exclusively secondary data from public domain sources, which were aggregated and contained no individual identifiers; therefore, it was exempt from review by a Research Ethics Committee, in accordance with Resolution No. 510 of April 7, 2016, of the Brazilian National Health Council.

#### Data availability statement:

the data used in this study are publicly available in the 2025 HIV/AIDS Epidemiological Bulletin of the Ministry of Health, in the Sinan and SIM systems, and in the IBGE population estimates accessed through DataSUS, as referenced in the Methods section.

## RESULTS

Between 2015 and 2023, 1,854 HIV cases, 2,026 AIDS cases, and 511 AIDS-related deaths were reported in the state of Tocantins. The average annual rates were 13.09 HIV cases per 100,000 inhabitants, 14.32 AIDS cases per 100,000 inhabitants, and 3.61 AIDS-related deaths per 100,000 inhabitants (Table 1).

**Table 1:** Notified HIV cases, AIDS cases, AIDS deaths, corresponding rates, and case-fatality rate, Tocantins, Brazil, 2015–2023.

Year	HIV n (%)	HIV detection rate*	AIDS n (%)	AIDS detection rate*	AIDS deaths n (%)	AIDS mortality rate*	Case-fatality rate (%) †
2015	185 (10.0)	12.30	228 (11.3)	15.17	54 (10.6)	3.59	23.7
2016	186 (10.0)	12.23	212 (10.5)	13.94	57 (11.2)	3.75	26.9
2017	185 (10.0)	12.03	256 (12.6)	16.65	53 (10.4)	3.45	20.7
2018	246 (13.3)	15.82	243 (12.0)	15.63	58 (11.4)	3.73	23.9
2019	202 (10.9)	12.84	177 (8.7)	11.25	48 (9.4)	3.05	27.1
2020	183 (9.9)	11.51	170 (8.4)	10.69	59 (11.5)	3.71	34.7
2021	220 (11.9)	13.69	222 (11.0)	13.81	64 (12.5)	3.98	28.8
2022	233 (12.6)	14.35	233 (11.5)	14.35	71 (13.9)	4.37	30.5
2023	214 (11.5)	13.04	285 (14.1)	17.37	47 (9.2)	2.86	16.5
**Total / Overall case-fatality rate**	**1,854 (100.0)**	**13.09** ‡	**2,026 (100.0)**	**14.32** ‡	**511 (100.0)**	**3.61** ‡	**25.2**

* Rates per 100,000 inhabitants, calculated based on intercensal population estimates from the Brazilian Institute of Geography and Statistics (*Instituto Brasileiro de Geografia e Estatística* – IBGE);

† Case-fatality rate calculated as the proportion of AIDS deaths relative to the total number of AIDS cases reported in the same year (%);

‡ Value corresponding to the mean of the annual rates over the analyzed period; n (%): absolute number and proportion relative to the total for the period.

Source: HIV/AIDS Epidemiological Bulletin 2025 (Ministry of Health); Notifiable Diseases Information System (*Sistema de Informação de Agravos de Notificação* – Sinan/DataSUS); Mortality Information System (*Sistema de Informações sobre Mortalidade* – SIM/DataSUS).

HIV detection rates fluctuated throughout the series, ranging from 11.51 per 100,000 inhabitants in 2020 to 15.82 per 100,000 inhabitants in 2018. AIDS detection rates ranged from 10.69 per 100,000 inhabitants in 2020 to 17.37 per 100,000 inhabitants in 2023. In turn, AIDS mortality rates showed less variation, ranging from 2.86 per 100,000 inhabitants in 2023 to 4.37 per 100,000 inhabitants in 2022 (Table 1).

In absolute numbers, both HIV and AIDS cases decreased in 2020, followed by an increase in subsequent years. The number of AIDS cases reached its highest value in the series in 2023 (285 cases), while the number of AIDS-related deaths ranged from 47 to 71 annually during the study period (Table 1).

The AIDS case-fatality rate ranged from 16.5 to 34.7%, with an overall value of 25.2%. The highest value was observed in 2020, whereas the lowest was recorded in 2023 (Table 1).

Temporal trend analysis using Prais-Winsten regression indicated stationary trends for all three evaluated indicators. The APC for the HIV detection rate was 1.1% (95%CI −1.5, 3.8; p=0.339); for the AIDS detection rate, 0.4% (95%CI −6.2, 7.5; p=0.887); and for the AIDS mortality rate, 0.4% (95%CI −2.4, 3.4; p=0.736) (Table 2).

**Table 2:** Temporal trends in HIV detection, AIDS detection, and AIDS mortality rates according to Prais–Winsten regression, Tocantins, Brazil, 2015–2023.

Indicator	APC (%)	95%CI	p-value	Durbin-Watson	Trend classification
HIV detection rate	1.1	−1.5; 3.8	0.339	2.402	Stationary
AIDS detection rate	0.4	−6.2; 7.5	0.887	1.196	Stationary
AIDS mortality rate	0.4	−2.4; 3.4	0.736	2.149	Stationary

APC: estimated annual percent change calculated using Prais–Winsten regression; 95%CI 95% confidence interval; Durbin–Watson: statistic used to assess serial autocorrelation of the residuals. The trend was classified as increasing when the 95%CI for the APC was entirely positive, decreasing when it was entirely negative, and stationary when it included zero.

Source: Notifiable Diseases Information System (*Sistema de Informação de Agravos de Notificação* – Sinan) and Mortality Information System (*Sistema de Informações sobre Mortalidade* – SIM)/DataSUS.

In the interrupted time-series analysis, with 2020 considered the interruption point, no significant immediate changes in level were observed for any of the evaluated indicators. The estimated level change was −2.14 cases per 100,000 inhabitants for the HIV detection rate (95%CI −7.63, 3.35; p=0.362), −4.39 for the AIDS detection rate (95%CI −10.77, 2.00; p=0.138), and 0.98 for the AIDS mortality rate (95%CI −0.85, 2.80; p=0.228) (Table 3).

**Table 3:** Interrupted time-series analysis of HIV detection, AIDS detection, and AIDS mortality rates (per 100,000 inhabitants), considering 2020 as the interruption point of the time series, Tocantins, Brazil, 2015–2023.

Indicator	Level change	95%CI	p-value	Post-interruption trend change (per year)	95%CI	p-value	Durbin-Watson	Classification
HIV detection rate	-2.14	-7.63; 3.35	0.362	0.06	-2.02; 2.13	0.946	2.748	Stationary
AIDS detection rate	-4.39	-10.77; 2.00	0.138	2.67	0.26; 5.09	0.036	2.257	Post-interruption increase
AIDS mortality rate	0.98	-0.85; 2.80	0.228	-0.11	-0.80; 0.58	0.709	2.390	Stationary

Interruption event defined as the year 2020, corresponding to the onset of the COVID-19 pandemic; Level change: immediate change in rates after the time-series interruption point; Trend change: annual percent change in rates during the post-interruption period; 95%CI 95% confidence interval; Durbin–Watson: statistic used to assess serial autocorrelation of the model residuals.

Source: Notifiable Diseases Information System (*Sistema de Informação de Agravos de Notificação* – Sinan) and Mortality Information System (*Sistema de Informações sobre Mortalidade* – SIM)/DataSUS.

Regarding the post-interruption trend, a significant increase was observed only in the AIDS detection rate, with an annual increase of 2.67 cases per 100,000 inhabitants (95%CI 0.26, 5.09; p=0.036). No significant change in the post-2020 trend was identified for the HIV detection rate (0.06; 95%CI −2.02, 2.13; p=0.946) or the AIDS mortality rate (−0.11; 95%CI −0.80, 0.58; p=0.709) (Table 3).

The Durbin–Watson statistics ranged from 2.257 to 2.748 in the interrupted time-series models, indicating no relevant serial autocorrelation in the residuals (Table 3).

**Figure 1:**
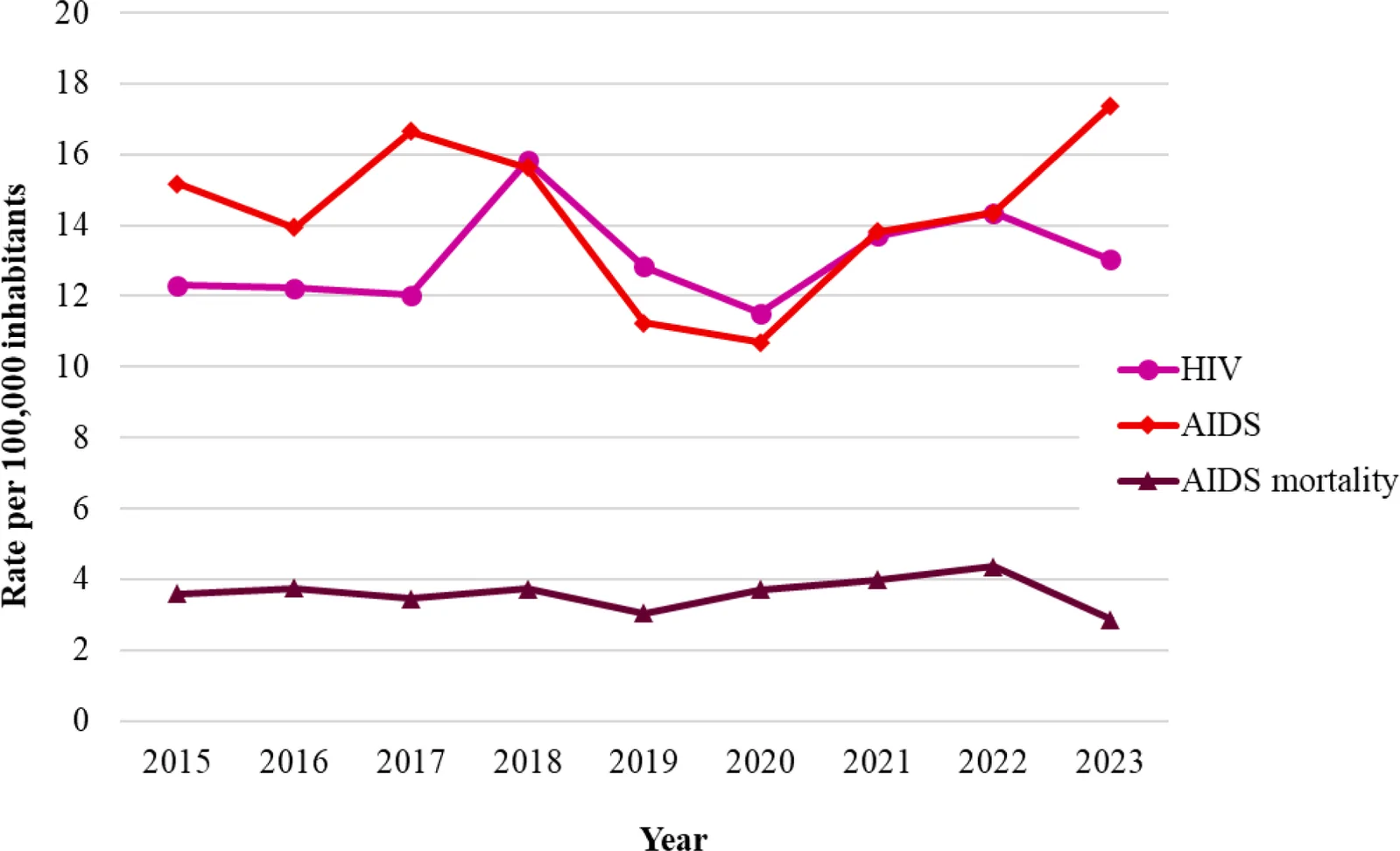
Temporal trends in HIV detection, AIDS detection, and AIDS mortality rates in Tocantins, Brazil, 2015–2023.

Source: Notifiable Diseases Information System (*Sistema de Informação de Agravos de Notificação* – Sinan), Medication Logistics Control System (*Sistema de Controle Logístico de Medicamentos* – Siclom), Laboratory Test Control System (*Sistema de Controle de Exames Laboratoriais* – Siscel), Mortality Information System (*Sistema de Informações sobre Mortalidade* – SIM), and Brazilian Institute of Geography and Statistics (*Instituto Brasileiro de Geografia e Estatística* – IBGE).

Figure 1 illustrates the temporal evolution of HIV detection, AIDS detection, and AIDS mortality rates in Tocantins between 2015 and 2023. HIV and AIDS detection rates decreased until 2020, followed by an increase in subsequent years, particularly for AIDS detection rates. In contrast, AIDS mortality rates showed smaller fluctuations, with no consistent change in the long-term trend.

## DISCUSSION

The results indicate stability in the long-term trends of HIV detection, AIDS detection, and AIDS mortality rates in Tocantins between 2015 and 2023. Meanwhile, the interrupted time-series analysis identified a significant increase in the trend of AIDS detection rates after the onset of the COVID-19 pandemic, whereas the other indicators remained unchanged. These findings suggest that the repercussions of the pandemic differed among the indicators analyzed.

The reduction in HIV and AIDS detection rates observed in 2020 coincided with the period when the COVID-19 pandemic had the greatest impact on health services. National and international studies have documented reductions in testing, decreases in the number of diagnoses, and the reorganization of healthcare activities during the first years of the health emergency^9,23,24^. The prioritization of care for COVID-19 cases, mobility restrictions, and changes in how services operated directly affected surveillance, diagnosis, and follow-up activities for people living with HIV^9,10,23,24^. In this context, the reduction observed in 2020 likely reflects, at least in part, temporary difficulties in accessing services and reduced diagnostic capacity.

A significant change in the trend of the AIDS detection rate after 2020 is the main finding of this study. This result suggests a progressive recovery of diagnostic and surveillance activities after the most critical phase of the pandemic, accompanied by the identification of cases that had remained undiagnosed during periods of greatest restriction in access to services. A similar phenomenon has been described in different countries and regions, where the resumption of healthcare activities was accompanied by an increase in the reporting of previously undiagnosed cases^9,23,24^. Thus, part of the increase observed after 2020 may reflect diagnostic delays and the recovery of the diagnostic capacity of health services.

Although the HIV detection rate also increased after 2020, the statistical analysis did not identify a significant change in the trend. This result suggests that the fluctuations observed throughout the series may be related to annual variability in reporting and fluctuations inherent in surveillance systems. The absence of a significant change reinforces the interpretation that HIV detection has remained relatively stable throughout the period analyzed.

A noteworthy finding was that the AIDS detection rate exceeded the HIV detection rate at the end of the historical series. In 2023, the AIDS detection rate was 17.37 cases per 100,000 inhabitants, whereas the HIV detection rate was 13.04 cases per 100,000 inhabitants. As these indicators are derived from different information systems and should be interpreted with caution, this pattern may reflect the persistence of late diagnoses, difficulties in timely access to testing, or a more pronounced recovery in AIDS case reporting after the pandemic period. Studies conducted in Brazil have shown that late diagnosis remains an important challenge for epidemic control, particularly in regions characterized by social inequalities and barriers to access to health services^1,2,8,9,25,26^.

The territorial characteristics of Tocantins should be considered when interpreting these results. The state has a large territorial area, municipalities with low population density, and inequalities in access to health services^20^. Studies conducted in the Northern region have shown that geographical barriers, limitations in healthcare infrastructure, and socioeconomic inequalities may influence access to diagnosis, treatment, and clinical care for people living with HIV^4–7,20,27^. In this context, state-level analyses contribute to understanding the regional dynamics of the epidemic and informing the planning of surveillance and care activities.

AIDS mortality showed fluctuations throughout the historical series; however, no significant increasing or decreasing trend was observed. This pattern suggests relative stability in the indicator and may reflect the maintenance of treatment and clinical follow-up activities throughout the period. National evidence demonstrates that expanded access to antiretroviral therapy and the consolidation of continuous care strategies have contributed to reducing the impact of the disease on mortality in recent decades^25,28,29^. The absence of a significant increase in mortality during the pandemic suggests that the disruptions observed in services did not translate into a measurable worsening of the indicator at the population level. However, this finding should be interpreted with caution, considering the possibility of delays in death reporting and the inherent limitations of health information systems.

The AIDS mortality rate reached its highest value in 2020, a year with the lowest number of cases reported in the historical series. This finding should be interpreted with caution because the case-fatality rate corresponds to the ratio between deaths and cases registered during the same period and may be influenced both by changes in the occurrence of deaths and by variations in case reporting. Thus, the increase observed in 2020 may reflect, in part, a temporary reduction in the identification and reporting of new cases during the initial phase of the pandemic^9,23,24,26^.

Among the study limitations, the ecological design and the use of secondary data from health information systems should be highlighted. The possibility of underreporting, reporting delays, incomplete databases, and differences in system coverage may influence the estimates presented. Furthermore, the use of aggregate data precludes inferences at the individual level and does not allow comprehensive control for factors potentially associated with the observed trends, such as changes in diagnostic coverage, population mobility, and programmatic changes that occurred throughout the period analyzed.

It should also be considered that the indicators were analyzed at the state level. Although this approach is appropriate for epidemiological monitoring, it does not allow for a comprehensive assessment of the inequalities existing among municipalities and health regions. Furthermore, HIV cases were obtained from the Ministry of Health's HIV/AIDS Epidemiological Bulletin, whose methodology is based on the integration of different information systems, making it impossible to assess the completeness and timeliness of the individual records used to construct this indicator. Additionally, the use of annual aggregates precludes the assessment of seasonal variations and reduces the ability to identify short-term effects associated with the pandemic.

Despite these limitations, this study contributes to understanding the recent dynamics of HIV/AIDS in Tocantins and to analyzing epidemiological indicators before and after the onset of the COVID-19 pandemic. The combination of Prais–Winsten regression and interrupted time-series analysis allows for the assessment of long-term trends and potential changes associated with the pandemic period.

The above reinforces the importance of continuously monitoring epidemiological indicators related to HIV and AIDS, particularly in contexts marked by territorial inequalities and challenges in access to health services. Although the overall trends remained stable during the period analyzed, the increase in the trend of the AIDS detection rate after 2020 suggests repercussions of the pandemic on the dynamics of disease diagnosis and surveillance. Strengthening surveillance activities, expanding access to testing, and ensuring timely monitoring of linkage to and retention in care remain essential for addressing the epidemic in the state of Tocantins and reducing late diagnoses of HIV infection.
