# Supplementary material for: Trends in HIV detection, AIDS detection, and ADIS mortality in Tocantins, Brazil, 2015–2023: Impact of the COVID-19 pandemic
Source: Rev Bras Epidemiol. 2026 Aug 28;29:e260050. doi: 10.1590/1980-549720260050 (PMC13524305; doi:10.1590/1980-549720260050)
Supplement: Translation

# Translation: Tendências da detecção de HIV, da detecção de aids e da mortalidade por aids no Tocantins, Brasil, 2015-2023: impacto da pandemia de COVID-19

**DOI:** 10.1590/1980-549720260050.2

**Authors:** Orcélia Pereira Sales, Edgar Merchan-Hamann

**Affiliations:** I Universidade de Brasília, Programa de Pós-Graduação em Ciências da Saúde – Brasília (DF), Brasil.

**Keywords:** HIV, AIDS, Estudos de Séries Temporais, COVID-19, Epidemiologia

## Abstract

**Objetivo:**

Analisar a evolução temporal dos indicadores epidemiológicos relacionados ao Vírus da Imunodeficiência Humana (HIV) e à Síndrome da Imunodeficiência Adquirida (Aids) no estado do Tocantins entre 2015 e 2023, e avaliar possíveis alterações associadas à pandemia de COVID-19.

**Métodos:**

Estudo ecológico de séries temporais com dados secundários de sistemas nacionais de informação em saúde. Foram calculadas taxas anuais por 100 mil habitantes. As tendências foram estimadas por regressão de Prais-Winsten, e as alterações associadas à pandemia por séries temporais interrompidas.

**Resultados:**

Entre 2015 e 2023, registraram-se 1.854 casos de HIV, 2.026 casos de aids e 511 óbitos por aids no Tocantins. As taxas médias anuais foram de 13,09 casos de HIV, 14,32 casos de aids e 3,61 óbitos por aids por 100 mil habitantes. A análise de Prais-Winsten indicou tendência estacionária para as taxas de detecção de HIV, detecção de aids e mortalidade por aids. Na análise de séries temporais interrompidas, não foram observadas mudanças imediatas significativas após 2020. Observou-se aumento significativo apenas da tendência da taxa de detecção de aids no período pós-interrupção (β = 2,67; IC95% 0,26; 5,09; p=0,036), enquanto as taxas de detecção de HIV e mortalidade por aids permaneceram estáveis.

**Conclusão:**

As tendências das taxas de detecção de HIV, detecção de aids e mortalidade por aids permaneceram estáveis no Tocantins entre 2015 e 2023. Entretanto, observou-se aumento da tendência da taxa de detecção de aids após o início da pandemia de COVID-19, sugerindo possíveis repercussões desse período sobre a dinâmica de diagnóstico e notificação da doença.

## INTRODUÇÃO

A infecção pelo Vírus da Imunodeficiência Humana (HIV) e a Síndrome da Imunodeficiência Adquirida (Aids) permanecem entre os principais desafios de saúde pública em escala global, mesmo após décadas de avanços no diagnóstico, no tratamento e na prevenção. A ampliação do acesso à terapia antirretroviral e às estratégias de prevenção combinada reduziu de forma expressiva a mortalidade e modificou o curso clínico da infecção. Persistem, contudo, desigualdades sociais e estruturais que condicionam o acesso aos serviços de saúde e a dinâmica de transmissão, de modo que a carga da doença continua distribuída de maneira desigual entre populações e territórios^1,2^.

No Brasil, a política de acesso universal e gratuito à terapia antirretroviral consolidou-se como referência internacional, mas a epidemia mantém marcada heterogeneidade territorial e social. As taxas de detecção e de mortalidade variam de modo substancial entre regiões e unidades federativas, acompanhando gradientes de renda, escolaridade e raça/cor que delimitam diferentes graus de vulnerabilidade. Determinantes socioeconômicos e estruturais influenciam tanto a exposição ao risco quanto o acesso oportuno à prevenção e ao tratamento, contribuindo para a persistência de iniquidades na resposta à epidemia^1–3^.

A região Norte concentra desafios adicionais para o enfrentamento de HIV/aids. A extensão territorial, a dispersão populacional e a oferta desigual de serviços de saúde dificultam o diagnóstico oportuno e a continuidade do cuidado, com repercussões sobre as tendências da detecção e da mortalidade. Estudos conduzidos na Amazônia brasileira descrevem trajetórias desfavoráveis da aids entre os estados, barreiras de acesso a ferramentas de prevenção como a profilaxia pré-exposição e padrões de mortalidade que acompanham as desigualdades regionais^4–6^.

O estado do Tocantins compartilha muitos dos desafios observados na região Norte, incluindo ampla extensão territorial, população distribuída em municípios de pequeno porte e desigualdades no acesso aos serviços de saúde^4,5,7^. Apesar disso, permanecem escassas as investigações que avaliam a evolução temporal dos indicadores relacionados a HIV/aids no estado, especialmente aquelas que consideram possíveis alterações associadas à pandemia de COVID-19^8,9^. Essa lacuna dificulta a compreensão da dinâmica recente da epidemia e limita a produção de evidências capazes de subsidiar o planejamento, o monitoramento e a avaliação das ações de vigilância, prevenção e cuidado.

A pandemia de COVID-19 representou um evento capaz de alterar significativamente a dinâmica dos serviços de saúde e dos sistemas de vigilância epidemiológica. A reorganização da rede assistencial, a priorização do atendimento aos casos de COVID-19 e as restrições de mobilidade impostas durante a emergência sanitária repercutiram sobre a oferta de testagem, o diagnóstico oportuno e o acompanhamento clínico das pessoas vivendo com HIV/aids^3,10,11^. Estudos realizados em diferentes contextos registraram redução dos diagnósticos, atrasos na vinculação ao cuidado e dificuldades de acesso aos serviços especializados durante os primeiros anos da pandemia^10–15^. No Brasil, observou-se redução expressiva dos registros de HIV e aids em 2020, fenômeno atribuído principalmente à diminuição da testagem e às mudanças na organização dos serviços de saúde^2,4,8^. Considerando as características territoriais e assistenciais da região Norte^4,5^, torna-se relevante investigar em que medida essas mudanças repercutiram sobre os indicadores epidemiológicos de HIV/aids no Tocantins.

Embora os impactos da pandemia sobre os referidos indicadores já tenham sido amplamente documentados em âmbito nacional e internacional, permanecem escassos os estudos que analisam, em escala estadual, as alterações nas tendências desses agravos, especialmente em contextos marcados por desigualdades territoriais, como o Tocantins. A ausência de análises que combinem a estimativa de tendências com a avaliação de pontos de interrupção limita a compreensão do comportamento recente da epidemia e a identificação de eventuais inflexões relacionadas ao período pandêmico.

Diante desse cenário, o presente estudo teve como objetivo analisar a evolução temporal dos indicadores epidemiológicos relacionados a HIV/aids no estado do Tocantins entre 2015 e 2023 e avaliar possíveis alterações associadas à pandemia de COVID-19.

## MÉTODOS

### Delineamento

Trata-se de um estudo ecológico de séries temporais^16^, baseado em dados secundários provenientes de sistemas nacionais de informação em saúde. O estudo foi conduzido de acordo com as recomendações da *Reporting of studies Conducted using Observational Routinely-collected health Data* (RECORD)^17^, extensão da declaração STROBE para pesquisas que utilizam dados coletados rotineiramente.

### Contexto

O estado do Tocantins, localizado na região Norte do Brasil, possui área territorial de 277.621 km² e população de 1.511.460 habitantes, segundo o Censo Demográfico de 2022 do Instituto Brasileiro de Geografia e Estatística (IBGE)^18^. O Índice de Desenvolvimento Humano Municipal (IDHM) foi de 0,731^19^. Administrativamente, o estado está organizado em oito Regiões de Saúde e duas Macrorregiões, totalizando 139 municípios^20^.

### Fonte de dados

Os dados referentes aos casos de HIV foram obtidos por meio do Boletim Epidemiológico HIV/Aids 2025^21^, publicado pelo Ministério da Saúde, que disponibiliza os registros de HIV provenientes da integração das bases do Sistema de Informação de Agravos de Notificação (Sinan), do Sistema de Controle de Exames Laboratoriais (Siscel) e do Sistema de Controle Logístico de Medicamentos (Siclom), segundo unidade federativa de residência e ano de diagnóstico. Foram extraídos os registros referentes ao estado do Tocantins para o período de 2015 a 2023. A inclusão de 5 anos anteriores ao início da pandemia de COVID-19 e de 4 anos posteriores permitiu ampliar a robustez das análises de tendência temporal e das séries temporais interrompidas.

Os dados referentes aos casos de aids foram obtidos por meio do Sinan, acessado pelo sistema TabNet do Departamento de Informação e Informática do Sistema Único de Saúde (DataSUS). As informações sobre mortalidade por aids foram extraídas do Sistema de Informação sobre Mortalidade (SIM), considerando os óbitos cuja causa básica foi classificada nos códigos B20 a B24 da Classificação Internacional de Doenças – 10ª revisão (CID-10). As estimativas populacionais utilizadas para o cálculo das taxas foram obtidas das projeções populacionais do IBGE, disponibilizadas por meio do DataSUS.

### Variáveis

Analisaram-se três indicadores epidemiológicos principais: taxa de detecção de HIV, taxa de detecção de aids e taxa de mortalidade por aids.

A taxa de detecção de HIV foi definida como o número de casos de HIV registrados em cada ano de diagnóstico por 100 mil habitantes, conforme metodologia adotada pelo Ministério da Saúde nos Boletins Epidemiológicos de HIV/Aids.

Já a taxa de detecção de aids correspondeu ao número de casos novos de aids notificados por 100 mil habitantes, enquanto a taxa de mortalidade por aids foi definida como o número de óbitos com causa básica registrada nos códigos B20 a B24 da CID-10 por 100 mil habitantes.

As taxas foram calculadas tomando-se como numerador o número anual de casos ou óbitos e, como denominador, a população residente estimada para o respectivo ano, segundo as estimativas populacionais do IBGE.

Também foram descritos os números absolutos de casos de HIV, aids e óbitos por aids registrados anualmente. Adicionalmente, foi calculada a letalidade por aids, definida como a razão entre o número de óbitos por aids e o número de casos de aids notificados no mesmo ano, expressa em percentual.

A letalidade por aids foi analisada exclusivamente de forma descritiva e não foi incluída nos modelos de tendência temporal, por representar uma razão entre eventos e não uma taxa populacional. Além disso, esse indicador é suscetível a oscilações decorrentes de pequenos números e à ausência de correspondência temporal entre o ano de notificação do caso e o ano de ocorrência do óbito.

### Análise dos dados

Realizou-se análise descritiva dos indicadores epidemiológicos, com cálculo de frequências absolutas, taxas anuais e letalidade^22^.

A tendência temporal das taxas de detecção de HIV, detecção de aids e mortalidade por aids foi estimada por regressão de Prais-Winsten, utilizando transformação logarítmica de base 10 das taxas. De acordo com os coeficientes estimados foram calculados a Variação Percentual Anual (VPA), os respectivos Intervalos de Confiança de 95% (IC95%) e os valores de p. As tendências foram classificadas como crescentes quando o IC95% da VPA foi totalmente positivo, decrescentes quando totalmente negativo e estacionárias quando o intervalo incluiu o valor nulo. A presença de autocorrelação serial dos resíduos foi avaliada por meio da estatística de Durbin-Watson.

Para a análise de séries temporais interrompidas, considerou-se o ano de 2020 como ponto de interrupção da série temporal, representando o início da pandemia de COVID-19. Os modelos de séries temporais interrompidas incluíram três componentes: uma variável de tempo, destinada a estimar a tendência pré-interrupção; uma variável indicadora de nível, utilizada para estimar alterações imediatas após 2020; e uma variável de interação entre tempo e período pós-interrupção, empregada para estimar alterações na tendência das séries após o início da pandemia.

A variável de nível foi codificada como 0 para o período anterior a 2020 e 1 para os anos subsequentes. A variável de tendência pós-interrupção foi construída atribuindo-se valor 0 aos anos anteriores à pandemia e valores crescentes aos anos posteriores, permitindo estimar a variação da tendência da série após a interrupção.

As análises foram realizadas no software R (versão 4.6.0), no ambiente *RStudio*, utilizando o pacote *prais* para os modelos de regressão de Prais-Winsten.

### Considerações sobre viés

Por se tratar de estudo ecológico baseado em dados secundários, os resultados estão sujeitos a subnotificação, atrasos de registro e diferenças na qualidade dos sistemas de informação. Mudanças na cobertura diagnóstica, na organização dos serviços e na vigilância epidemiológica, especialmente durante a pandemia de COVID-19, podem ter influenciado os indicadores analisados. Além disso, a utilização de dados agregados impede inferências em nível individual, estando os resultados sujeitos à falácia ecológica.

### Considerações éticas

O estudo utilizou exclusivamente dados secundários de domínio público, agregados e sem identificação individual, estando dispensado de apreciação por Comitê de Ética em Pesquisa, conforme a Resolução n.° 510, de 7 de abril de 2016, do Conselho Nacional de Saúde.

#### Declaração de disponibilidade de dados:

os dados utilizados neste estudo são de domínio público e estão disponíveis no Boletim Epidemiológico HIV/Aids 2025 do Ministério da Saúde e nos sistemas Sinan, SIM e nas estimativas populacionais do IBGE, acessados via DataSUS, conforme referenciado na seção Métodos.

## RESULTADOS

Entre 2015 e 2023, foram registrados 1.854 casos de HIV, 2.026 casos de aids e 511 óbitos por aids no estado do Tocantins. As taxas médias anuais corresponderam a 13,09 casos de HIV por 100 mil habitantes, 14,32 casos de aids por 100 mil habitantes e 3,61 óbitos por aids por 100 mil habitantes (Tabela 1).

**Tabela 1:** Casos notificados de HIV, casos de aids, óbitos por aids, taxas correspondentes e letalidade, Tocantins, Brasil, 2015-2023.

Ano	HIV n (%)	Taxa de detecção de HIV*	aids n (%)	Taxa de detecção de aids*	Óbitos por aids n (%)	Taxa de mortalidade por aids*	Letalidade (%) †
2015	185 (10,0)	12,30	228 (11,3)	15,17	54 (10,6)	3,59	23,7
2016	186 (10,0)	12,23	212 (10,5)	13,94	57 (11,2)	3,75	26,9
2017	185 (10,0)	12,03	256 (12,6)	16,65	53 (10,4)	3,45	20,7
2018	246 (13,3)	15,82	243 (12,0)	15,63	58 (11,4)	3,73	23,9
2019	202 (10,9)	12,84	177 (8,7)	11,25	48 (9,4)	3,05	27,1
2020	183 (9,9)	11,51	170 (8,4)	10,69	59 (11,5)	3,71	34,7
2021	220 (11,9)	13,69	222 (11,0)	13,81	64 (12,5)	3,98	28,8
2022	233 (12,6)	14,35	233 (11,5)	14,35	71 (13,9)	4,37	30,5
2023	214 (11,5)	13,04	285 (14,1)	17,37	47 (9,2)	2,86	16,5
**Total / Letalidade global**	**1854 (100,0)**	**13,09** ‡	**2026 (100,0)**	**14,32** ‡	**511 (100,0)**	**3,61** ‡	**25,2**

* Taxas por 100 mil habitantes, calculadas com base nas estimativas populacionais intercensitárias do Instituto Brasileiro de Geografia e Estatística (IBGE);

† Letalidade calculada como a proporção de óbitos por aids em relação ao total de casos de aids notificados no mesmo ano (%);

‡ Valor correspondente à média das taxas anuais no período analisado; n (%): número absoluto e proporção em relação ao total do período.

Fonte: Boletim Epidemiológico HIV/Aids 2025 (Ministério da Saúde); Sistema de Informação de Agravos de Notificação (Sinan/DataSUS); Sistema de Informações sobre Mortalidade (SIM/DataSUS).

As taxas de detecção de HIV apresentaram oscilações ao longo da série, variando de 11,51, em 2020, a 15,82 por 100 mil habitantes em 2018. As taxas de detecção de aids oscilaram entre 10,69, em 2020, e 17,37 por 100 mil habitantes em 2023. Por sua vez, a taxa de mortalidade por aids apresentou menor amplitude de variação, com valores entre 2,86 por 100 mil habitantes em 2023 e 4,37 por 100 mil habitantes em 2022 (Tabela 1).

Em números absolutos, observou-se redução dos casos de HIV e de aids em 2020, seguida de aumento nos anos subsequentes. Os casos de aids atingiram o maior valor da série em 2023 (285 casos), enquanto os óbitos por aids oscilaram entre 47 e 71 registros anuais no período analisado (Tabela 1).

A letalidade por aids variou entre 16,5 e 34,7%, com valor global de 25,2%. O maior valor foi observado em 2020, enquanto o menor foi registrado em 2023 (Tabela 1).

A análise de tendência temporal por regressão de Prais-Winsten indicou comportamento estacionário para os três indicadores avaliados. A VPA da taxa de detecção de HIV foi de 1,1% (IC95% −1,5; 3,8; p=0,339); da taxa de detecção de aids, 0,4% (IC95% −6,2; 7,5; p=0,887); e da taxa de mortalidade por aids, 0,4% (IC95% −2,4; 3,4; p=0,736) (Tabela 2).

**Tabela 2:** Tendência temporal das taxas de detecção de HIV, detecção de aids e mortalidade por aids segundo regressão de Prais-Winsten, Tocantins, Brasil, 2015-2023.

Indicador	VPA (%)	IC95%	p-valor	Durbin-Watson	Classificação da tendência
Taxa de detecção de HIV	1,1	−1,5; 3,8	0,339	2,402	Estacionária
Taxa de detecção de aids	0,4	−6,2; 7,5	0,887	1,196	Estacionária
Taxa de mortalidade por aids	0,4	−2,4; 3,4	0,736	2,149	Estacionária

VPA: variação percentual anual estimada por regressão de Prais-Winsten; IC95% intervalo de confiança de 95%; Durbin-Watson: estatística utilizada para avaliação de autocorrelação serial dos resíduos. A classificação da tendência foi definida como crescente quando o IC95% da VPA foi totalmente positivo, decrescente quando totalmente negativo e estacionária quando incluiu o valor zero.

Fonte: Sistema de Informação de Agravos de Notificação (Sinan) e Sistema de Informações sobre Mortalidade (SIM)/DataSUS.

Na análise de séries temporais interrompidas, considerando o ano de 2020 como ponto de interrupção da série temporal, não foram observadas mudanças imediatas significativas de nível para nenhum dos indicadores avaliados. A mudança de nível estimada foi de −2,14 casos por 100 mil habitantes para a taxa de detecção de HIV (IC95% −7,63; 3,35; p=0,362), −4,39 para a taxa de detecção de aids (IC95% −10,77; 2,00; p=0,138) e 0,98 para a taxa de mortalidade por aids (IC95% −0,85; 2,80; p=0,228) (Tabela 3).

**Tabela 3:** Análise de séries temporais interrompidas das taxas (por 100 mil habitantes) de detecção de HIV, detecção de aids e mortalidade por aids, considerando 2020 como ponto de interrupção da série temporal, Tocantins, Brasil, 2015-2023.

Indicador	Mudança de nível	IC95%	p-valor	Mudança de tendência pós-interrupção (por ano)	IC95%	p-valor	Durbin-Watson	Classificação
Taxa de detecção de HIV	-2,14	-7,63; 3,35	0,362	0,06	-2,02; 2,13	0,946	2,748	Estacionária
Taxa de detecção de aids	-4,39	-10,77; 2,00	0,138	2,67	0,26; 5,09	0,036	2,257	Aumento pós-interrupção
Taxa de mortalidade por aids	0,98	-0,85; 2,80	0,228	-0,11	-0,80; 0,58	0,709	2,390	Estacionária

Evento de interrupção definido como o ano de 2020, correspondente ao início da pandemia de COVID-19; Mudança de nível: variação imediata nas taxas após o ponto de interrupção da série; Mudança de tendência: variação percentual anual nas taxas no período posterior à interrupção; IC95% intervalo de confiança de 95%; Durbin-Watson: estatística utilizada para avaliação de autocorrelação serial dos resíduos do modelo.

Fonte: Sistema de Informação de Agravos de Notificação (Sinan) e Sistema de Informações sobre Mortalidade (SIM)/DataSUS.

Em relação à tendência após a interrupção, observou-se aumento significativo apenas para a taxa de detecção de aids, com acréscimo anual de 2,67 casos por 100 mil habitantes (IC95% 0,26; 5,09; p=0,036). Para a taxa de detecção de HIV não foi identificada alteração significativa da tendência após 2020 (0,06; IC95% −2,02; 2,13; p=0,946), assim como para a taxa de mortalidade por aids (−0,11; IC95% −0,80; 0,58; p=0,709) (Tabela 3).

Os valores da estatística de Durbin-Watson variaram entre 2,257 e 2,748 nos modelos de séries temporais interrompidas, indicando ausência de autocorrelação serial relevante dos resíduos (Tabela 3).

A Figura 1 ilustra a evolução temporal das taxas de detecção de HIV, detecção de aids e mortalidade por aids no Tocantins entre 2015 e 2023. Observa-se redução das taxas de detecção de HIV e aids até 2020, seguida de aumento nos anos subsequentes, especialmente para a taxa de detecção de aids. Em contraste, a mortalidade por aids apresentou oscilações de menor magnitude e sem alteração consistente de tendência ao longo da série.

**Figura 1:** Tendência temporal das taxas de detecção de HIV, detecção de aids e mortalidade por aids no Tocantins, Brasil, 2015-2023.

Fonte: Sistema de Informação de Agravos de Notificação (Sinan), Sistema de Controle Logístico de Medicamentos (Siclom), Sistema de Controle de Exames Laboratoriais (Siscel), Sistema de Informações sobre Mortalidade (SIM) e Instituto Brasileiro de Geografia e Estatística (IBGE).

## DISCUSSÃO

Os resultados indicaram estabilidade das tendências de longo prazo das taxas de detecção de HIV, detecção de aids e mortalidade por aids no Tocantins entre 2015 e 2023. Entretanto, a análise de séries temporais interrompidas identificou aumento significativo da tendência da taxa de detecção de aids após o início da pandemia de COVID-19, enquanto os demais indicadores permaneceram estáveis. Esses achados sugerem que as repercussões da pandemia diferiram entre os indicadores analisados.

A redução das taxas de detecção de HIV e de aids observada em 2020 coincide com o período de maior impacto da pandemia de COVID-19 sobre os serviços de saúde. Estudos nacionais e internacionais documentaram diminuição da testagem, redução do número de diagnósticos e reorganização das atividades assistenciais durante os primeiros anos da emergência sanitária^9,23,24^. A priorização do atendimento aos casos de COVID-19, as restrições de mobilidade e as mudanças no funcionamento dos serviços repercutiram diretamente sobre as ações de vigilância, diagnóstico e acompanhamento das pessoas vivendo com HIV^9,10,23,24^. Nesse contexto, a redução observada em 2020 provavelmente reflete, ao menos em parte, dificuldades temporárias de acesso aos serviços e diminuição da capacidade diagnóstica.

A mudança significativa da tendência da taxa de detecção de aids após 2020 constitui o principal achado deste estudo. Esse resultado sugere recuperação progressiva das atividades de diagnóstico e vigilância após a fase mais crítica da pandemia, acompanhada pela identificação de casos que permaneceram sem diagnóstico durante os períodos de maior restrição de acesso aos serviços. Fenômeno semelhante foi descrito em diferentes países e regiões, onde a retomada das atividades assistenciais foi acompanhada pelo aumento dos registros de casos previamente não diagnosticados^9,23,24^. Assim, parte do crescimento observado após 2020 pode refletir atraso diagnóstico e recuperação da capacidade de detecção dos serviços de saúde.

Embora a taxa de detecção de HIV também tenha apresentado aumento após 2020, a análise estatística não identificou alteração significativa da tendência. Esse resultado sugere que as oscilações observadas ao longo da série podem estar relacionadas à variabilidade anual dos registros e às flutuações inerentes aos sistemas de vigilância. A ausência de mudança significativa reforça a interpretação de que o comportamento da detecção de HIV permaneceu relativamente estável no estado ao longo do período analisado.

Um achado que merece destaque foi a maior taxa de detecção de aids em relação à taxa de detecção de HIV ao final da série histórica. Em 2023, a taxa de detecção de aids foi de 17,37 casos por 100 mil habitantes, enquanto a taxa de detecção de HIV foi de 13,04 casos por 100 mil habitantes. Embora esses indicadores sejam derivados de sistemas de informação distintos e devam ser interpretados com cautela, esse padrão pode refletir persistência de diagnósticos tardios, dificuldades de acesso oportuno à testagem ou recuperação mais intensa dos registros de aids após o período pandêmico. Estudos realizados no Brasil têm demonstrado que o diagnóstico tardio permanece um desafio importante para o controle da epidemia, especialmente em regiões marcadas por desigualdades sociais e barreiras de acesso aos serviços de saúde^1,2,8,9,25,26^.

As características territoriais do Tocantins devem ser consideradas na interpretação desses resultados. O estado apresenta ampla extensão territorial, municípios com baixa densidade populacional e desigualdades no acesso aos serviços de saúde^20^. Estudos realizados na região Norte têm demonstrado que barreiras geográficas, limitações da infraestrutura assistencial e desigualdades socioeconômicas podem influenciar o acesso ao diagnóstico, ao tratamento e ao acompanhamento clínico das pessoas vivendo com HIV^4–7,20,27^. Nesse contexto, análises em escala estadual contribuem para compreender a dinâmica regional da epidemia e subsidiar o planejamento das ações de vigilância e cuidado.

A mortalidade por aids apresentou oscilações ao longo da série histórica, porém sem tendência significativa de aumento ou redução. Esse comportamento sugere relativa estabilidade do indicador e pode refletir a manutenção das ações de tratamento e acompanhamento clínico ao longo do período. Evidências nacionais demonstram que a ampliação do acesso à terapia antirretroviral e a consolidação das estratégias de cuidado contínuo contribuíram para reduzir o impacto da doença sobre a mortalidade nas últimas décadas^25,28,29^. A ausência de aumento significativo da mortalidade durante e após a pandemia sugere que as interrupções observadas nos serviços não se traduziram em agravamento mensurável desse indicador em nível populacional. Contudo, esse resultado deve ser interpretado com cautela, considerando a possibilidade de atrasos no registro dos óbitos e as limitações inerentes aos sistemas de informação em saúde.

A letalidade por aids apresentou seu maior valor em 2020, ano que registrou o menor número de casos notificados da série histórica. Esse resultado deve ser interpretado com cautela, pois a letalidade corresponde à razão entre óbitos e casos registrados em um mesmo período e pode ser influenciada tanto por alterações na ocorrência dos óbitos quanto por variações na notificação dos casos. Dessa forma, o aumento observado em 2020 pode refletir, em parte, a redução temporária da identificação e notificação de novos casos durante a fase inicial da pandemia^9,23,24,26^.

Entre as limitações do estudo destacam-se o delineamento ecológico e a utilização de dados secundários provenientes de sistemas de informação em saúde. A possibilidade de subnotificação, atrasos de registro, incompletude das bases e diferenças na cobertura dos sistemas pode influenciar as estimativas apresentadas. Além disso, a utilização de dados agregados impede inferências em nível individual e não permite controlar integralmente fatores potencialmente associados às tendências observadas, como mudanças na cobertura diagnóstica, mobilidade populacional e alterações programáticas ocorridas ao longo do período analisado.

Também deve ser considerado que os indicadores foram analisados em escala estadual. Embora essa abordagem seja adequada para o monitoramento epidemiológico, ela não permite captar integralmente as desigualdades existentes entre municípios e regiões de saúde. Além disso, os casos de HIV foram obtidos por meio do Boletim Epidemiológico HIV/Aids do Ministério da Saúde, cuja metodologia resulta da integração de diferentes sistemas de informação, o que impossibilita a avaliação individual da completude e oportunidade dos registros utilizados na construção desse indicador. Adicionalmente, a agregação anual dos dados impossibilitou a avaliação de variações sazonais e reduziu a capacidade de identificar efeitos de curto prazo associados à pandemia.

Apesar das limitações, o estudo contribui para a compreensão da dinâmica recente de HIV/aids no Tocantins ao analisar indicadores epidemiológicos antes e após o início da pandemia de COVID-19. A combinação da regressão de Prais-Winsten com séries temporais interrompidas permitiu avaliar tendências de longo prazo e possíveis alterações associadas ao período pandêmico.

Os achados reforçam a importância do monitoramento contínuo dos indicadores epidemiológicos relacionados ao HIV e à aids, especialmente em contextos marcados por desigualdades territoriais e desafios de acesso aos serviços de saúde. Embora as tendências gerais tenham permanecido estáveis no período analisado, o aumento da tendência da taxa de detecção de aids após 2020 sugere repercussões da pandemia sobre a dinâmica de diagnóstico e vigilância da doença. O fortalecimento das ações de vigilância, a ampliação do acesso à testagem e o monitoramento oportuno da vinculação e da permanência no cuidado permanecem fundamentais para o enfrentamento da epidemia no estado do Tocantins e para a redução dos diagnósticos tardios da infecção pelo HIV.
